# Using Advisory Boards to Develop a Faith-Based Toolbox to Support African American Families Facing Dementia

**DOI:** 10.1093/geroni/igab046.1233

**Published:** 2021-12-17

**Authors:** Fayron Epps

**Affiliations:** Emory University, Atlanta, Georgia, United States

## Abstract

For this project, we are designing and testing the feasibility of employing components of a Faith-based Home Activity Toolbox (Faith-HAT). The goal of this NIH stage 1 intervention development project is to go beyond the four walls of the church to find ways to meet the spiritual needs of persons living with moderate and severe dementia “where they are” to help them remain religiously and spiritually engaged. This mixed-methods project is designed in 2 phases: (a) developing a prototype Faith-HAT and (b) testing the feasibility and exploring preliminary effectiveness. To successfully conduct this project, we have included a community advisory board of church leaders, caregivers, and persons living with dementia as members of the research design team to advise on the design and implementation of the Faith-HAT. Brainstorming workshops with the board are used to ensure the research is meeting the needs of the African American families affected by dementia.

